# Preparation Methods and Functional Characteristics of Regenerated Keratin-Based Biofilms

**DOI:** 10.3390/polym14214723

**Published:** 2022-11-04

**Authors:** Ruirui Wang, Hui Tong

**Affiliations:** Department of Applied Chemistry, College of Chemistry and Chemical Engineering, Qinghai Normal University, 38 Wusi West Road, Xining 810008, China

**Keywords:** keratin, biofilm, preparation, characteristics, application prospect

## Abstract

The recycling, development, and application of keratin-containing waste (e.g., hair, wool, feather, and so on) provide an important means to address related environmental pollution and energy shortage issues. The extraction of keratin and the development of keratin-based functional materials are key to solving keratin-containing waste pollution. Keratin-based biofilms are gaining substantial interest due to their excellent characteristics, such as good biocompatibility, high biodegradability, appropriate adsorption, and rich renewable sources, among others. At present, keratin-based biofilms are a good option for various applications, and the development of keratin-based biofilms from keratin-containing waste is considered crucial for sustainable development. In this paper, in order to achieve clean production while maintaining the functional characteristics of natural keratin as much as possible, four important keratin extraction methods—thermal hydrolysis, ultrasonic technology, eco-friendly solvent system, and microbial decomposition—are described, and the characteristics of these four extraction methods are analysed. Next, methods for the preparation of keratin-based biofilms are introduced, including solvent casting, electrospinning, template self-assembly, freeze-drying, and soft lithography methods. Then, the functional properties and application prospects of keratin-based biofilms are discussed. Finally, future research directions related to keratin-based biofilms are proposed. Overall, it can be concluded that the high-value conversion of keratin-containing waste into regenerated keratin-based biofilms has great importance for sustainable development and is highly suggested due to their great potential for use in biomedical materials, optoelectronic devices, and metal ion detection applications. It is hoped that this paper can provide some basic information for the development and application of keratin-based biofilms.

## 1. Introduction

Enormous amounts of keratin-containing waste (e.g., hair, wool, feather, and so on) are produced by various industries, such as textiles, food, leather, technology, and so on. According to statistics, the world produces over 2.5 million tonnes of wool, greater than 40 million tons of hair, and more than 65 million tonnes of feathers every year [1,2,3,4]. This waste fur and hair, which contains about 90% keratin, remains a rich, yet unexploited bio-resource for bio-film production [5]. At present, most of this keratin-containing waste is treated in landfills, leading to a range of environmental pollution [6]. As such, the recycling, development, and application of keratin-containing waste provides an important means to address environmental pollution and energy shortages [7]. Keratin can easily be extracted from natural fur and hair, such as wool, chicken feathers, hog hairs, rabbit hairs, human hairs, and so on [8,9,10,11,12]. In this line, the development of keratin-based functional materials has become the key to solving waste fur and hair pollution.

Keratin is an important fibrous structural protein present in waste fur and hair [13], which possesses unique physicochemical characteristics. Keratin is a natural biopolymer that is distinguished from other proteins by its high cysteine content (7–13%) [14,15]. Keratin is a type of intermediate filament found in the cytoskeleton of eukaryotic cells and contains high levels of certain amino acids, such as alanine, glycine, serine, and valine while having lower levels of lysine, tryptophan, and methionine [16,17,18,19,20]. Its molecular structure contains many active groups (e.g., carboxyl, amino, thiol, and hydroxyl groups) which can be modified. These chemical groups on keratin molecules act with each other to produce non-covalent intra- and inter-molecular bonds, thus forming macromolecular aggregates of keratin. The non-covalent bonds mainly include disulphide bonds, hydrogen bonds, hydrophobic bonds, and ionic bonds. Keratin is divided into two categories: alpha-keratin (α-helix) and beta-keratin (β-sheets) [21]. Both alpha- and beta-keratin are found in birds and reptiles while alpha-keratin is primarily seen in mammals [22]. As a primary component of wool, feathers, hair, and other materials, keratin is frequently used in the biomedical field, due to its biodegradability and compatibility with living tissue [23,24,25]. Keratin extracted from keratin-containing waste was studied for cosmetics, feed additives, and biomedical applications, among other fields. As disulphide bonds can promote intermolecular cross-linking, keratin is considered an excellent raw material for biofilms [26]. Keratin-based biofilm provides an effective alternative to conventional polystyrene film. Keratin-based biofilms are gaining substantial interest due to their unique molecular composition and excellent characteristics, such as good biocompatibility, high biodegradability, appropriate adsorption, rich renewable sources, and so on [27]. At present, keratin-based biofilms are widely considered a good option for various applications and the development of keratin-based biofilms from keratin-containing waste is crucial for sustainable development.

## 2. Novel Extraction Methods of Keratin

**Figure 1:**
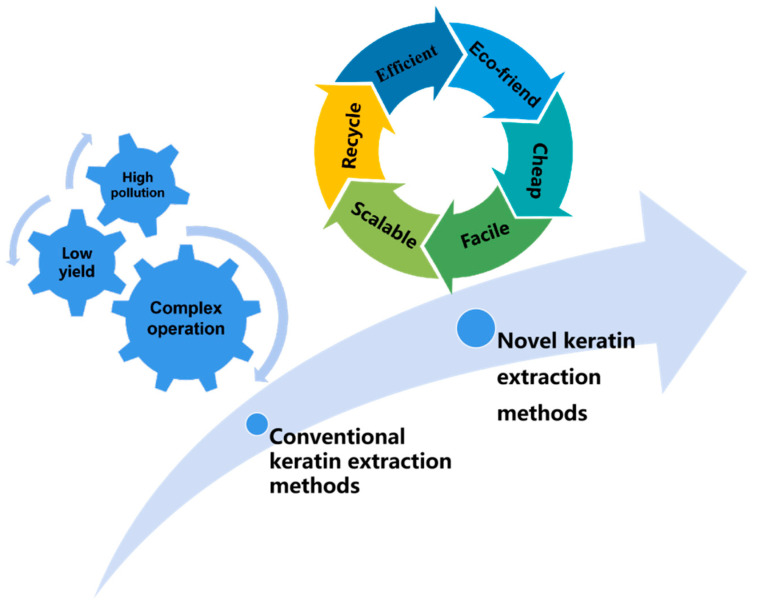
Development direction of keratin extraction methods.

Disulphide bonds are the key to maintaining the structural stability of keratin molecules [28]. As such, the disruption of inter- and intra-molecular disulphide bonds in natural fur and hair is the most critical step during keratin extraction [29]. To extract keratin, highly concentrated toxic chemicals (acid and alkali) are customarily used to destroy the inter- and intra-molecular disulphide bonds in the fur and hair. As can be seen from Table 1, reported extraction methods have all used chemical or enzymatic methods to cleave these disulphide bonds. The original ordered structure of the keratin is destroyed and a randomly disordered structure is formed as the fur and hair disintegrate during keratin extraction. In general, as the amount of chemical reagent increases, the degree of destruction of non-covalent bonds increases, which then improves the extraction efficiency of keratin. As shown in Table 1, conventional keratin extraction methods include acidic hydrolysis, alkaline hydrolysis, sulphitolysis hydrolysis, reduction, oxidation, enzymatic hydrolysis, and so on. These keratin extraction methods all require a large number of environmentally harmful solvents, such as sodium sulphide, metabisulphite, 2-mercaptoethanol, and so on. These methods create some problems such as high pollution, time-consuming, high cost, complex operation, and associated health and environmental risks, which are not suitable for the economic scaling up of keratin production [30]. Meanwhile, the formation of quantitatively disordered structures may yield regenerated keratin with significant performance defects, such as poor mechanical properties, weak flexibility, and inferior thermal property. Researchers have dedicated significant efforts to develop novel, reliable, and scalable keratin extraction methods from keratin-containing waste, with the aim of achieving clean production while maintaining the functional characteristics of natural keratin as much as possible. This section mainly reviews the novel keratin extraction methods, as shown in Figure 1. 

### 2.1. Thermal Hydrolysis

Tasaki [1] has proposed a novel method for keratin extraction from hog hairs using a two-step heating thermal hydrolysis process (THP). The first heating temperature is at or above keratin’s denaturing temperature. The first stage can sufficiently dilate and loosen the network structure composed of keratin molecular chains. Meanwhile, the hydrogen bonds maintaining the keratin structure are disrupted. The second heating temperature is between 100 and 220 °C. At this stage, water molecules are further diffused into the keratin fibre network structure, in order to cleave the disulphide bonds; thus, the keratins are dissolved into the water. The action of the second stage is consistent with that of the chemical reductant 2-mercaptoethanol on hairs. This THP process does not require chemicals and will not promote environmental and health risks. The thermal hydrolysis technology is clean, safe, and eco-friendly; however, the extraction conditions are harsh and the keratin obtained through this method has low molecular weight and is a mixture of amino acids and polypeptides. Avoiding excessive hydrolysis of the keratin during extraction and improving the stability of the extraction process is key to ensuring the scalable production of thermal hydrolysis technology. 

### 2.2. Ultrasonic Technology

Ultrasonic technology is widely used in the biomedical field. Strong mechanical vibration and acoustic cavitation disrupt the intra- and inter-molecular interactions of keratin, causing the accelerated dissolution of keratin during ultrasound extraction. Meanwhile, acoustic cavitation produces a transient high temperature in the extraction medium, which temperature promotes the production of secondary reducing radicals, thus enhancing the mass transfer efficiency. Azmi et al. [73] investigated feather keratin extraction by combining ultrasound with [BMIM]Cl Ionic liquids, where the intervention of ultrasound significantly shortened the extraction time. The test indicated that ultrasonication does not disrupt the backbone structure of keratin. Ultrasonic techniques can shorten the extraction time and reduce the extraction temperature. As an eco-friendly and promising method to extract keratin, it can be controlled according to the ultrasonic irradiation time and the acoustic power. Ultrasonic technology is simple to operate, fast, and efficient; however, to implement the extraction process, special equipment is required. Therefore, at present, it can only be used as an auxiliary technology to conventional keratin extraction methods. In this regard, reducing the cost of the related equipment is key to ensuring the scalable production of ultrasonic technology. 

### 2.3. Eco-Friendly Solvent System 

The use of eco-friendly, recyclable solvent systems has opened new perspectives for the extraction of keratin. Using an eco-friendly solvent system to disrupt the inter- and intra-molecular interactions and disulphide bonds in native keratin allows for keratin extraction. The eco-friendly solvent system must provide a high enough yield and maximally retain the secondary structure of keratin; that is, the entire extraction process should not cause excessive hydrolysis of keratin.

Cassoni et al. [74] investigated a novel eco-friendly method using a commercial abstergent, Mistolin^®^ HTG 50, to extract keratin from pig hair. The abstergent is composed of less than 5% non-ionic surfactants and 5–15% sodium hydroxide. The extracted keratin solution is then separated and purified using an ultrafiltration membrane (SD 3838 BS03-S, Iberlact, Madrid, Spain, MWCO: 10 kDa). The pilot test results indicated that the purity of keratin obtained using this method ranged up to 70%. The use of a biodegradable non-ionic surfactant as a keratin extractant can simplify the extraction process, avoid harmful reagents, and reduce the extraction cost, which is suitable for industrial production.

Ionic liquids (ILs) are substances made up of organic/inorganic anions and bulky cations in liquid form at relatively low temperatures [2]. The structures of cations or anions in ILs determine their properties. In principle, up to 10^18^ ILs can be designed by changing cations and anions [75]. As designer solvents, ILs are considered green solvents for keratin extraction. ILs have high thermal stability and low vapor pressure [76,77,78]. ILs can disrupt the hydrogen bonds and disulphide bonds in keratin molecules, thus promoting keratin dissolution.

Ji et al. [79] developed a novel simple and eco-friendly method to extract duck feather keratin using the imidazole ionic liquid [Bmim]Cl. Their results indicated that the keratin was highly efficiently extracted from duck feathers (75.1% yield of keratin). The extraction solvent was filtered and subjected to reduced pressure distillation, and the water and volatile impurities were removed. The ILs can be recycled three times during the keratin extraction. Liu et al. [80] used a series of DBN(1,5-diazabicyclo[4.3.0]-non-5-ene)-based ILs to extract wool keratin. In particular, 8 wt% goat wool was completely dissolved in [DBNE]DEP ILs at 393 K for 3 h. The crystallinity and the thermal stability of the keratin regenerated from [DBNE]DEP was higher than that from [DBNH]OAc ILs and [Emim]DEP ILs. Furthermore, [DBNE]DEP can be recycled more than five times. Zhang et al. [81] compared the capability of different ionic liquids to disrupt disulphide bonds. The results showed that the capability of anions to disrupt disulphide bonds in [Bmim]^+^ based ILs was ordered as follows: [OAc]^−^ >Cl^−^ > Br^−^ > [DBP]^−^ > [H2PO4]^−^. Meanwhile, the capability of anions to disrupt disulphide bonds in [Emim]^+^-based ILs was as follows: [OAc]^−^ > Cl^−^ > [DEP]^−^ > [DMP]^−^. The capability of cations to disrupt disulphide bonds in Cl^−^-based ILs was as follows: [Emim]^+^ > [Amim]^+^ ≈ [Bmim]^+^ > [Hmim]^+^ > [BPy]^+^ > [P4444]^+^ > [N4444]^+^. Overall, [Emim]OAc ILs and [Bmim]OAc ILs had stronger disulphide bond disrupting capability, while quaternary ammonium-based ILs and phosphonium-based ILs had weaker disrupting capability. The change in side chains of imidazolium-based ILs had little effect on disulphide bond disrupting capability. It is worth noting that, in order to avoid excessive disruption of the keratin microstructure during ionic liquid extraction, ILs with moderate disulphide bond disrupting capability (70–80%) should be selected as keratin extractants. To enhance the extraction efficiency and extraction yield, researchers have used some auxiliary methods in the process of extracting keratin with ILs [82,83,84]; for example, Feroz et al. [83] reported rapid keratin extraction from wool using ILs (tetrabutylphosphonium hydroxide, TBPH; and choline hydroxide) assisted by probe sonication. The new technology required no additional heating, and the results showed that the probe sonication technique enables rapid and efficient extraction of wool keratin. TBPH showed a higher extraction efficiency, compared to choline hydroxide. As innovative keratin extractants, ILs-based extraction is safer, more effective, and more environmentally friendly than conventional extraction (see Table 1) [85]; however, ILs are relatively more expensive and the preparation conditions are more demanding, which greatly hinders relevant industrial applications. At present, reducing the extraction cost is still difficult, in terms of extracting keratin with ILs. As ILs have good thermal stability, they can be recycled; therefore, the recycling of ILs on a large scale urgently needs to be solved.

Deep eutectic solvents (DESs) possess similar physical properties to ILs, such as low vapor pressure, incombustibility, and so on. DESs are mainly composed of hydrogen bond donors (e.g., organic acids, polyols, amides, urea, and sugars) and hydrogen bond receptors (e.g., quaternary ammonium salts, amino acids, and metal ions), constituting a networked system of hydrogen bonds. The most significant physical property of DESs is their lower melting point than their components. DESs possess high extraction ability and good solubilisation strength [86]. Several DESs were studied for regenerative keratin extraction, including a choline chloride–urea DES, choline chloride–oxalic acid DES, choline–ethylene glycol DES, choline–citric acid DES, and so on [87,88,89,90,91]. Among these, the choline chloride–urea DES is currently the most commonly used DES for keratin extraction. Jiang et al. [92] extracted wool keratin using chloride–urea DES. As a hydrogen bond donor, urea can interact with chloride, thereby reducing the melting point of the mixture. The results showed that 1 g DES could completely dissolve 35.1 mg wool when kept at 130 °C for 5 h. The regenerated wool keratin retained its long peptide chains. The choline chloride–urea DES is an effective solvent for keratin extraction. DESs have low toxicity, degradability, and sustainability characteristics. Their preparation process is simple and atomically economical [93]. DESs are the most promising alternative to conventional keratin extractants. DESs are easier, cheaper, and have more sustainable chemistry to extract keratin than ILs. However, strong hydrogen bonding leads to DESs with high viscosity (>100 mPa·s), which hinders the mass transfer efficiency between the DES and fur or hair during extraction. Therefore, reducing the viscosity of the DESs is key to improving the keratin extraction efficiency and achieving large-scale production.

### 2.4. Microbial Decomposition

The use of aerobic and anaerobic micro-organisms to catalyse the decomposition of natural fur and hair is a new idea for extracting keratin. Yeo et al. [94] digested chicken feathers with an extremely thermophilic bacterium *Fervidobacterium islandicum* AW-1, in order to extract keratin. The strain possesses a variety of proteolytic enzymes that can decompose hair. The extracted keratin inhibited the expression of UVB-induced MMP-1 and MMP-13 in human dermal fibroblasts; thus, the extracted keratin can be used as a cosmeceutical peptide to prevent skin aging. Microbial decomposition requires mild extraction conditions. The whole extraction process of microbial catalytic decomposition is simple, safe, and pollution-free, making it a promising method for extracting bioactive keratin. Keratin resulting from the microbial decomposition of fur and hair has antioxidant properties and the ability to inhibit ACE and dipeptidyl peptidase IV activities [70]; however, microbial decomposition can easily destroy the peptide bonds in the keratin structure, resulting in low molecular weight of the extracted keratin.

These novel keratin extraction methods can solve the problems of low keratin extraction yield and complicated extraction processes, to a certain extent; however, there are still some problems, such as high production cost and poor reproducibility, which hinder the large-scale production of regenerated keratin in this way. Even good-quality regenerated keratin still has the problems of brittle texture and low strength. The properties of the regenerated keratin are not comparable to those of the natural keratin. As a result, the microbial extraction of keratin is still in the laboratory research stage. It is necessary to further study the relationship between the keratin extraction conditions and the microstructure of regenerated keratin, which is expected to provide the theoretical foundation for optimising keratin processing and accelerating the industrial utilisation of keratin production and suitable recycled keratin materials.

## 3. Preparation Method of Keratin-Based Biofilms

**Figure 2:**
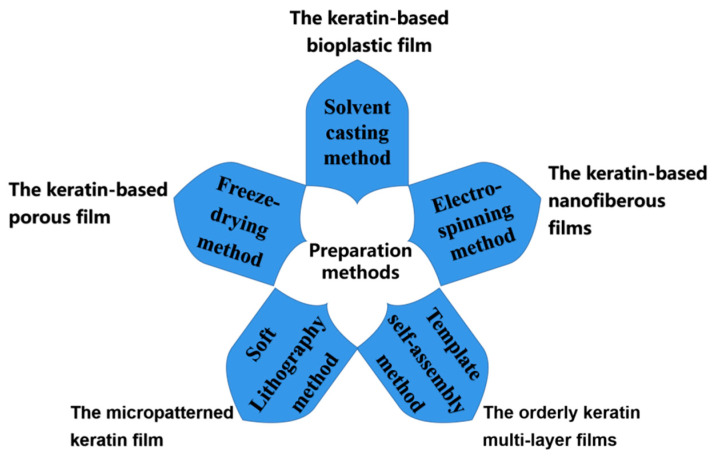
Keratin-based biofilm preparation methods.

This section mainly reviews the main preparation methods for keratin-based biofilms, as shown in Figure 2.

### 3.1. Solvent Casting Method

Solvent casting is an effective, low-cost, and simple-to-operate method for the development of keratin-based functional films. However, as the backbone of the natural fur and hair is heavily damaged during extraction, pure keratin films prepared using the solvent casting method may have poor mechanical properties, seriously hindering the application of the pure keratin film. To improve the mechanical properties of keratin-based biofilms, it is necessary to introduce other chemicals to modify the keratin during casting. At present, the main modification methods include grafting, blending, and cross-linking modification.

Grafting modification employs a polymerisation reaction to introduce functional side groups onto the keratin molecular chains. Li et al. [7] adopted lipoic acid, a natural antioxidant, to graft modify wool keratin. Lipoic acid has two structures, open-ring or closed-ring, where a redox reaction can transform the two structures into each other. In the process of keratin modification, the ring structure of lipoic acid is opened, following which the thiol groups on keratin molecules are grafted. Their results indicated that lipoic acid grafting modification might regulate the structure of the keratin-based biofilm, and the mechanical properties of the resulting keratin-based biofilm were increased significantly. Grafting modification can successfully introduce active groups onto the keratin molecules, significantly improving the mechanical properties of keratin-based biofilms. However, ensuring a high grafting rate and uniformity of grafting products is still the key to grafting modification. Blending modification involves the selection of certain functional polymers, such as thermoplastic polyurethane, polyvinyl alcohol, and polyethylene oxide, to mix with keratin in order to form macroscopic homogeneous films [95,96,97]. Blending modification not only can maintain the inherent characteristics of keratin film but can also make up for the defects of keratin film through the synergistic effect between keratin and the functional polymer(s). The blending modification process is carried out synchronously with the film formation process and, thus, does not require complicated post-treatment steps. The modifiers do not destroy the keratin structure or its function. To improve the performance of the keratin-based biofilm, cross-linkers are necessary during solvent casting. Traditional chemical cross-linkers include aldehydes, carbodiimides, epoxy compounds, and acyl azides [98]. Intra- or inter-molecular cross-linking of keratin can significantly enhance the mechanical strength and reduce the antigenicity of the keratin-based biofilm; however, the obtained cross-linked films may be highly cytotoxic [99]. It should be noted that the introduced cross-linkers must be non-toxic, feasible, and efficient. In this line, biological enzymatic catalytic cross-linkers are considered to be ideal candidates for improving the physical properties of keratin-based biofilms [100,101].

Compared with other film-forming methods, the advantages of solvent casting methods are the simple manufacturing process, the convenience of operation, and the uniform film structure. The disadvantages of this method are that it is time consuming and can easily introduce impurities. In order to improve the properties of keratin-based biofilms, many natural polymers as modifiers, such as sodium alginate, gelatine, chitosan, cellulose, pullulan, and silk fibroin may be introduced into the casting solution [102,103,104,105,106,107,108].

### 3.2. Electrospinning Method

In electrospinning, the keratin solution is sprayed under a strong electric field to obtain keratin filaments. The keratin filaments are then deposited and solidified onto a collecting device to form nanofibrous films. These keratin nanofibrous films have a large specific area, high aspect ratio, and high porosity. At the same time, they are uniform and their surface treatment is simple. As the keratin solution is sprayed into keratin filaments under a strong electric field during electrospinning, accurate control of the viscosity of the keratin solution is key to the success of electrospinning. The lower viscosity of the keratin solution will decrease the strength of the spinning, resulting in spinning failure; in contrast, the higher viscosity of the keratin solution will increase the resistance of spinning, also resulting in spinning failure. Keratin solution alone is difficult to spin, due to its poor viscosity [109]; as such, it is usually necessary to add polymers or prepare high molecular weight keratin, in order to adjust the viscosity of the spinning fluid. Researchers have investigated the compatibility of keratin with various polymers in co-spinning, and successfully developed various types of keratin-based nanofibrous films [110,111]. Aluigi et al. [112] prepared keratin-based nanofibrous films using a mixture of wool keratin and polyamide (PA6) as the electrospinning stock solution. The results demonstrated that keratin and PA6 had similar molecular structures. Due to rapid solvent evaporation during electrospinning, keratin-based nanofibrous films with phase-free separation and homogeneous structure can be obtained. The content of keratin is proportional to the viscosity of the spinning fluid and inversely proportional to the diameter of the keratin-based fibrous film. Ye et al. [23] prepared high molecular weight wool keratin through transglutaminase catalytic cross-linking. The obtained wool keratin and poly (3-hydroxybutyric acid-3-hydroxyvaleric acid) (PHBV) were electrospun to form nanofibrous films, following which silver nanoparticles were generated on the keratin-based nanofibrous films by in situ bioreduction. Their results demonstrated that the keratin-based nanofibrous films had good antibacterial properties, high biocompatibility, and strong wound-healing ability.

Compared with other film-forming methods, electrospinning is the most effective method to prepare keratin-based nanofibrous films with controllable pore size, which may range from tens of nanometres to several micrometres. Keratin-based nanofibrous films formed using electrospinning are endowed with various attractive attributes, such as good filtration performance, high biocompatibility, and low trans-epidermal water loss [113,114]. However, electrospinning often requires the use of organic solvents, which are difficult to recover due to the particularity of the operation. Current research has mainly focused on the addition of harmless biodegradable natural polymers and environmentally friendly solvents, in order to regulate the viscosity of the keratin-based spinning fluid.

### 3.3. Template Self-Assembly Method

The stability of keratin depends on certain factors, such as the degree of ionisation, amino acid sequence, the density of charges, and their distribution along the peptide chain. A large number of cysteine residues containing sulphur endow keratin with good self-assembly ability. Keratin is a desirable building block for template self-assembly. When the charged keratin solution is coated on the substrate surface, the cation and anion will be alternately deposited on the substrate surface due to electrostatic attraction. By inducing and regulating the self-assembly arrangements, regular and orderly keratin multi-layer films can be produced on the substrate surface.

Yang et al. [115] employed wool keratin as a novel building component, where iodoacetic acid was used to protect the thiol groups of the keratin. Then, a layer-by-layer (LbL) self-assembly method was used to create keratin/polyelectrolyte multi-layer membranes on a quartz matrix. Depending on the pH of the solution, wool keratin could be utilised as a polycation (pH < 3.8) or polyanion (pH > 3.8) for the construction of (PDDA/keratin)_n_ or PDDA+(PAA/keratin)_n_ multi-layer films. The driving force of the LBL self-assembly process was the electrostatic interaction. Overall, keratin enhanced the biocompatibility of the multi-layer films. Ferraris et al. [116] coupled wool keratin to a titanium surface by molecular grafting and thick coatings. Their results indicated that plasma surface activation enhanced the bonding ability between keratin and the titanium substrate. The titanium surfaces modified with wool keratin had good mechanical stability and cell adhesion, and the keratin film on the titanium surface improved the biocompatibility of the titanium implants.

The template self-assembly method enables the keratin to spontaneously form regular supramolecular structures on the substrate surface through non-covalent interactions. Keratin decorates the substrate surface in the template self-assembly method, allowing the substrate to obtain good biocompatibility and no immune toxicity. Template self-assembly can regulate the thickness of keratin films and endow the substrate surface with certain functional characteristics. Compared with other film-forming methods, the template self-assembly method restricts the keratin structure to the template surface and guides the self-assembly behaviour of keratin. The template self-assembly method can be implemented under mild conditions and is suitable for various substrates. Keratin-functionalised implants can reduce the risk of inflammation/infection development. There is an increasing demand for novel keratin-based self-assembly films in biotechnology and medicine applications. At present, enhancing the mechanical adhesion and the long-term stability between keratin and the substrate is a key problem to be solved in self-assembly.

### 3.4. Freeze-Drying Method

Keratin solution forms a distinctly interconnected porous network structure when freeze-dried. The freeze-drying process only relies on the sublimation of ice crystals in the keratin solution [117]; as such, there is no need to introduce toxic chemical reagents in the freeze-drying process, making the operation process clean and facile.

Postai et al. [118] prepared a keratin porous sponge film by freeze-drying. To improve the insolubility of the keratin porous sponge film for application in water treatment, oxidised sucrose was used as a cross-linker. The maximum adsorption capacities of the keratin porous sponge film for Azure A and methyl orange were 0.063 and 0.037 mmol/g, respectively. Zhuang et al. [119] also prepared chicken keratin porous and flexible sponge film using a freeze-drying method. Interestingly, they used the insoluble residues from the feather keratin extraction as a film-forming material. The insoluble residues were produced during the extraction of feather keratin by sodium hydrogen sulphite reduction. The maximum Cr(VI) uptake capacity of the sponge films was about 148.8 mg/g, and the removal rate of PM_10_ was 98.3%. Kakkar et al. [120] prepared a novel keratin-based porous film containing chitosan and gelatine using a freeze-drying method. Their results indicated that the keratin-based porous film prepared by freeze-drying possessed good thermal stability, good mechanical properties, and strong water absorption capacity. Ramadoss et al. [121] manufactured a gelatine/keratin (GK) film by freeze-drying and showed that it displays good mechanical strength, high porosity (366 ± 49 µm), sustained drug release, and good hemocompatibility. Therefore, the GK film can be used as a natural bandage for wound healing.

The freeze-drying method is the simplest method to prepare a keratin-based film with a porous microstructure, compared with other film-forming methods. The structure of the film can be regulated by changing the concentration of the film-forming fluid and the freezing time [122,123,124]. Freeze-drying methods have better controllability than electrospinning methods. The keratin-based biofilms prepared by freeze-drying typically possess high porosity and suitable pore size to promote cell adhesion, proliferation, and diffusion. These keratin-based biofilms can be used as scaffolds in vivo, in order to simulate the cell micro-environment, thus playing a key role in tissue engineering [125]. Unfortunately, pure keratin biofilms prepared by freeze-drying are characterised by brittleness and weak mechanical strength [126]. Therefore, as with electrospinning, natural polymers are usually required to enhance the mechanical strength of the resulting keratin-based film. It should be emphasised that the introduced polymers must be non-toxic, harmless, and able to cooperate with keratin.

### 3.5. Soft Lithography Method

Soft lithography is a facile, environmentally friendly, and functionally diverse patterned technology. The “Top-down” etching technology promotes the construction of protein micro- and nanostructures. Unlike traditional photolithography methods, soft lithography does not destroy the original structure and function of keratin. Zhu et al. [127] developed a soft lithography approach to fabricate high-precision spatially patterned keratin film. In order to gain the photoactive wool keratin precursor, the wool keratin was modified using the chemical reagent 2-isocyanatoethyl methacrylate (IEM). Then, the wool keratin film with patterned microstructures was obtained by soft lithography. Their results showed that soft lithography was a facile method to acquire wool keratin film with surface microstructures and good optical properties.

As compared with other film-forming methods, soft lithography methods provide the ability to regulate and control the surface molecular structure and biologically related complex molecules. Moreover, this type of method is particularly suitable for constructing complex 3D microstructures on irregular curved substrates [128]. The micropatterned keratin film can be used to manufacture channel structures suitable for microfluidics and to control cell growth. However, soft lithography methods have many problems to be solved, such as requiring expensive equipment and the films being prone to contamination [129].

## 4. Functional Properties of Keratin-Based Films

**Figure 3:**
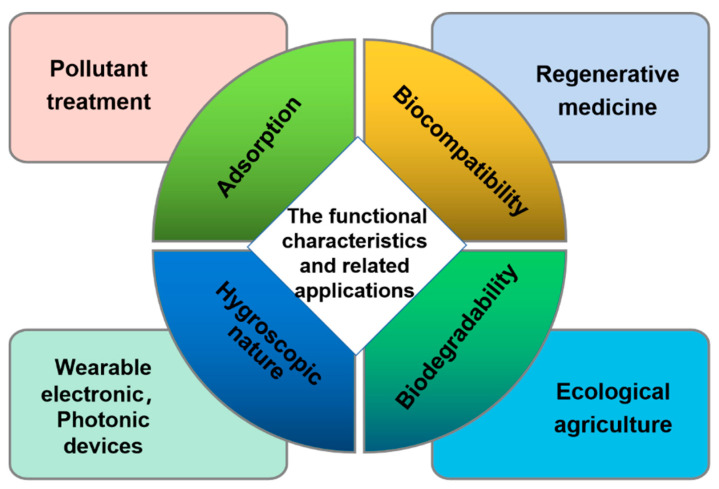
The functional characteristics and related applications of keratin-based biofilms.

This section mainly reviews the functional characteristics of keratin-based biofilms, as detailed in Figure 3.

### 4.1. Biocompatibility

Cell binding motifs, such as leucine–aspartic acid–valine (LDV), glutamic acid–aspartic acid–serine (EDS), and arginine–glycine–aspartic acid (RGD) binding residues are generally present in keratin [130,131]. Furthermore, the extracted keratin has a strong self-assembly ability and can form regular and ordered structures for the regulation of cell behaviour [132]. This can promote cell–matrix interactions and provide a good living environment for cells. The keratin-based biofilm can create a favourable 3D matrix displaying strong cytocompatibility or may be used as a culture dish to promote the adhesion, proliferation, and migration of cells in biomedicine.

Navarro et al. [133] used keratin biofilms cross-linked by dityrosine for guided tissue regeneration (GTR). The keratin biofilms provided a cross-linked network that allows for the diffusion of molecules, such as media nutrients and growth factors, thus regulating the transport channels. Keratin biofilms may regulate tissue growth by slowing rapidly proliferating cells and allowing slower cells to migrate and reconstruct specialised microstructures.

Keratin-based biofilms provide an opportunity for the physiological regeneration of skin functions and aesthetic reconstructions of complex features. Due to its excellent biocompatibility, keratin-based biofilm can provide cells with a good living environment (i.e., similar to the ECM), stimulate collagen expression, accelerate fibroblast regeneration, and regulate growth rates. Keratin-based biofilm has exhibited significant potential for use as a chronic wound dressing in the field of regenerative medicine, due to its good biocompatibility in vivo and its excellent ability to accelerate wound healing [134,135,136]. Meanwhile, keratin-based biofilm was also used as a transferable film for the reconstruction of the corneal surface [46,137,138]. A significant amount of clinical research is still required to expand the clinical application of keratin-based biofilms in the future.

### 4.2. Biodegradability

Keratin is a smart protein with high biodegradability, high sulphur content, and high nitrogen content, compared to other proteins. The proteins and amino acids in keratin-based films can be used as nutrients for plant growth [139].

Gaidau et al. [19] employed alkaline or alkaline enzymatic processes to hydrolyse wool waste. Their results showed that the keratin hydrolysate possesses approximately 9.2% cysteine sulphur, as compared to the original wool. Their keratin has a natural resistance to bacteria or fungi, as well as biostimulant activity for seed and plant growth. The keratin hydrolysate can also be used as a biostimulant for natural plant growth. Keratin-based biofilms possess high biodegradability, compared to native fur and hair. The degraded keratin can serve as a C and N source for soil micro-organisms [140,141,142]; in particular, the degraded keratin can strengthen the soil respiration of micro-organisms, as well as adjust the pH and expand the cation exchange capacity of the soil. Based on this, keratin-based biofilms have great potential for conversion into biofertilisers. These biofertilisers not only provide nutrition to the land, in order to improve the growth rate and yield of plants but also can reduce the soil damage caused by the application of artificial chemicals and excessive chemical fertilisers [143]. The high biodegradability of keratin-based biofilms opens up a new direction for the manufacture of environmentally friendly and nutritional mulch films in ecological agriculture [144]. However, at present, in order to meet the mechanical properties required by agricultural mulch film, some non-degradable substances are still used in the preparation of keratin-based biofilms. Moreover, the preparation cost of keratin biofilm is high. Therefore, the biggest challenge in the development of keratin-based agricultural mulch films is reducing their cost and avoiding the introduction of non-degradable substances in the film formation system as much as possible.

### 4.3. Hygroscopic Nature

A large number of hydrophilic groups (e.g., amino, carboxyl, and hydroxyl groups) are present in keratin. The random coil and α-helical structures are predominant in the keratin film, and the keratin film has a highly hydrophilic nature. The hydrogen and hydroxyl ions (H^+^ and OH^−^) formed by water dissociation move under the action of an applied electric field. Keratin chains tend to form a crystalline α-helical structure and yield an H-bond water network with amino acid residues on its side chain. This is also accompanied by proton transfer under high humidity conditions [145]. Meanwhile, the structure of the keratin film is porous and rough. Therefore, the keratin film can be used as a humidity-sensing material for electrochemical sensors.

Hamouche et al. [146] used wool keratin film as a humidity-sensitive element in order to prepare a planar capacitive humidity sensor. Their results indicated that the obtained keratin-based capacitive humidity sensors displayed high sensitivity and low hysteresis. The response and recovery time of these sensors were reasonably fast: for interdigitated and spiral electrodes, the response time was about 36 s and 30 s, respectively, while the recovery time was about 55 s and 51 s. The sensing mechanism of a capacitive humidity sensor is the dielectric change in the keratin film caused by vapor adsorption and desorption. Humidity-sensitive capacitors are generally made using polymer film capacitors. Commonly used polymer materials are polystyrene, polyimide, polyaniline, acetate fibre, and so on [147,148,149,150,151]. When the ambient humidity changes, the dielectric constant of the humidity-sensitive capacitor changes, such that its capacitance also changes, in a manner directly proportional to the relative humidity. Keratin film is a suitable candidate for humidity-sensing applications, due to its low surface roughness, high transparency, excellent biocompatibility, suitable flexibility, high moisture absorption, low-temperature processing, and low cost. Natali et al. [145] developed a keratin-based flexible sensor for surveying humidity flow. Keratin film not only served as the active ionic electrolyte for water ion sensing, but also the flexible and insoluble substrate in this sensor. Glutaraldehyde and glycerol were employed to improve insolubility and to realise the repeated use of the keratin-based sensor. Their results showed that the keratin film modified by selective chemical doping provides a bendable, biocompatible, and transparent substrate for sensing applications. The linear relationship between the output electrical signals of the keratin-based bipolar (KBs) devices and the ambient humidity level was certified. The keratin-based multielectrode array system can offer a dependable, stable, and calibrated amperometric output signal.

In short, we should exploit the hygroscopic nature of keratin to manufacture multi-functional keratin-based films with conformability, reliability, and biocompatibility. Based on this, the further development of keratin-based wearable electronic and photonic devices is expected to become a research hotspot in the biomedical field.

### 4.4. Adsorption

A large number of active groups, such as amino, hydroxyl, and carboxyl groups, on the molecular chain and the special backbone of keratin, confer a more appropriate adsorption capacity to keratin-based biofilm, compared with other low-cost absorbing materials [152]. The polar amino acid residues contained in keratin can bind to cationic substances (e.g., heavy metals or dyes) [153,154], such as aspartic acid, glutamic acid, arginine, cystine, cysteine, and other amino acids, which account for more than 38% of the total amino acids [155]. Modified keratin has shown strong adsorption capacity for the metals As, Cd, Cu, V^V^, K, Co, Ni, Zn, and Cr^VI^ [156]. Toxic water pollutants, such as heavy metals, dyes, and antibiotics, can be fixed in the network structure of the keratin-based film through complex reactions [118,157,158,159]. Qian et al. [160] prepared a cellulose nanocrystal (CNC)/keratin composite film and investigated its blue KN-R dye adsorption behaviour. Their results demonstrated that the 2.5%CNC/keratin film possessed high dye adsorption capacity. The dye adsorption mechanism of the keratin-based film is monolayer adsorption. The dye adsorption process consisted of Langmuir’s adsorption isotherm model and a pseudo-second-order model. Aluigi et al. [161] evaluated the Cu^2+^ ion-adsorption capacity of a wool keratin/polyamide 6 nanofiber film prepared by electrospinning. Their results indicated that the free carboxyl groups of the keratin side-chain enabled chelation with Cu^2+^ ions; as such, the keratin-based nanofiber film showed a high adsorption capacity for Cu^2+^ ions, which was directly proportional to the specific surface area of the nanofiber film. Therefore, keratin-based fibre films are suitable candidates for sewage disposal. Song et al. [152] designed a mechanically reinforced keratin/silk fibre composite film. Their results showed that the keratin-based biofilm possessed high adsorption capacity and removal efficiency for reactive brilliant blue KN-R and the keratin/silk composite film could be recycled.

Keratin, derived from agricultural and industrial waste, when used to create keratin-based biofilms, can provide economic, effective, and eco-friendly absorbent systems for wastewater treatment or air pollution control in fields such as dyeing, metal plating, mining operations, and so on. Keratin-based biofilms can also be used for metal ion detection. Furthermore, the excellent adsorption capacity of keratin-based biofilms can also improve the electrical conductivity of keratin, leading to many prospects in the field of biotechnology.

## 5. New Development Direction of Keratin-Based Biofilm

As keratin-based biofilms can be readily obtained from keratin-containing waste (e.g., hair, wool, feather, and so on), the high-value conversion of keratin-containing waste into keratin-based biofilms has great importance for sustainable development. Keratin-based biofilms have great potential in biomedical materials, optoelectronic devices, and metal ion detection applications, among many others. Future research in this area should focus on the following aspects:

(1) Due to the different sources and complex structures, keratin is difficult to extract and purify, which increases the costs related to producing keratin-based biofilms. In the future, we should continue to develop cost-effective, time-efficient, and eco-friendly keratin extraction methods, in order to achieve efficient large-scale production of keratin-based biofilms. Only in this way can the waste and environmental pollution related to fur and hair be solved;
(2) The structure–activity relationship between the structural and functional properties during the preparation of keratin-based biofilms should be further investigated. Precise regulation of the degradation rate of natural keratin-based biofilms to achieve the controlled release of drugs comprises a key difficulty to be resolved in the future;
(3) The unique properties of keratin-based biofilms should be fully exploited in order to further expand their application range.

## Figures and Tables

**Table 1:** Conventional keratin extraction methods.

Extract Methods	Solvent(s)	Process Conditions	Extraction Theory	Merit	Defect	Ref.
Acidic hydrolysis	HCl solution	Wool was placed in 4 M HCl solution and incubated at 95 °C.	RSSR’ + OH^−^↔RSH + R’SH	High efficiency, Low molecular weight, Simple and practicable, Low cost.	Significant acid pollution Strong acids destroy the keratin structure and some important amino acids, such as tryptophan, asparagine, arginine, serine, and glutamine. Other amino acids are racemised.	[30,31]
Alkaline hydrolysis	NaOH solution	Duck feathers were immersed in 2% (wt) NaOH solution at 60–70 °C for 2 h.	RSSR’ + OH^−^↔RSSO^−^ + R’H	Keratin amino acid damage during alkaline hydrolysis is substantially lower, as compared to acidic hydrolysis.	Cannot be recycled, Difficult to handle, Time-consuming, Low yield.	[32,33,34,35,36,37]
Reduction	2-mercaptoethanol	Human hair was immersed in the Shindai solution of 25 mM Tris-HCl, 2.6 M thiourea, 5 M urea, 5% (*v*/*v*) 2-mercaptoethanol, and then incubated at 50 °C for 72 h.	RSSR’ + 2HOCH_2_CH_2_SH↔RSH + R’SH + HOCH_2_CH_2_S-SCH_2_CH_2_OH	High yield, High efficiency, High molecular weight keratin, The keratin structure is undamaged.	Toxic, Harmful, Expensive, multi-steps, Cannot be recycled, Not industrially viable.	[38,39,40,41,42,43,44,45,46,47,48]
Sulphitolysis	Metabisulphite	Wool was placed in a solution of urea (8 M) and metabisulphite (0.5 M), adjusted to pH 6.5, and agitated at 65 °C for 2 h.	RSSR’ + SO_3_^2−^↔RS^−^ + R’S-SO_3_^−^	Highly efficient, Low molecular weight keratin, Short extraction time.	Low solubility, High extract temperature.	[22,49,50,51,52,53]
	Sodium sulphide	Human hair was immersed in 0.125 M sodium sulphide solution and incubated at 40 °C for 4 h.	Na_2_S + H_2_O↔2Na^+^ + HS^−^ + OH^−^ RSSR’ + HS^−^↔RSH + R’S-S^−^	Efficient, Economical, High yield, Low extract temperature, The antioxidant potency and the secondary structure of keratin are saved.	Time-consuming, Low solubility.	[54,55,56,57,58,59]
	L-cysteine	Chicken feathers were placed in the solutions of 8 M urea and 0.165 M of L-cysteine, adjusted to pH 10.5 using NaOH (2 M).	RSSR’ + 2^−^SCH_2_CH(NH_2_)COO^−^↔RS^−^ + R’S^−^ + ^−^OOC(NH_2_)CHCH_2_S-SCH_2_CH(NH_2_)COO^−^	Eco-friendly, More crystalline structure, The structure of keratin is better saved. Low-cost, Mild treatment conditions, L-cysteine is non-toxic.	Low yield.	[60,61,62,63,64,65]
	Sodium bisulphite	Chicken feathers were added to a solution of 1.78% NaOH and 0.5% NaHSO_3_, and incubated at 87 °C for 111 min.	RSSR’ + HSO_3_^−^↔RSH + R’S- SO_3_^−^	Highly effective, Low-cost, Moderate molecular weight keratin, Moderate solubility, Non-toxic.	High extract temperature.	[9,66]
oxidation	Peracetic acid	Human hair was placed in 2.5% (*w*/*v*) peracetic acid solution overnight at room temperature.	RSSR’ + 3O^2−^↔RSO_3_^−^ + R’SO_3_^−^	Low molecular weight keratin, Mild extraction conditions, Good solubility.	Low yield.	[67,68,69]
Enzymatic hydrolysis	Keratinases	Wool was immersed in 100 mL of a water solution containing 100 kU of keratinase and vigorously stirred for 24 h at 50 °C.	The peptide bonds on the keratin backbone are interrupted using an enzymatic lytic response.	Bioactive keratins, Eco-friendly, Few species alterations, Safe, Mild treatment conditions, No contaminants.	Expensive, Low efficiency, Time-consuming.	[23,70,71,72]

## Data Availability

Not applicable.
